# Time Efficiency, Reliability, and User Satisfaction of the Tooth Memo App for Recording Oral Health Information: Cross-Sectional Questionnaire Study

**DOI:** 10.2196/56143

**Published:** 2024-04-10

**Authors:** Palinee Detsomboonrat, Pagaporn Pantuwadee Pisarnturakit

**Affiliations:** 1 Department of Community Dentistry Faculty of Dentistry Chulalongkorn University Bangkok Thailand

**Keywords:** capability, health survey, oral health, mobile apps, personal health information, PHI, satisfaction, tooth, teeth, oral, dental, dentist, dentistry, data entry, data collection, mHealth, mobile health, app, apps, applications, periodontal, survey, questionnaire, questionnaires

## Abstract

**Background:**

Digitalizing oral health data through an app can help manage the extensive data obtained through oral health surveys. The Tooth Memo app collects data from oral health surveys and personal health information.

**Objective:**

This study aims to evaluate the evaluate the time efficiency, reliability, and user satisfaction of the Tooth Memo app.

**Methods:**

There are 2 sections in the Tooth Memo app: oral health survey and personal oral health record. For the oral health survey section of the Tooth Memo app, different data entry methods were compared and user satisfaction was evaluated. Fifth-year dental students had access to the oral health survey section in the Tooth Memo app during their clinical work. The time required for data entry, analysis, and summary of oral health survey data by 3 methods, that is, pen-and-paper (manual), Tooth Memo app on iOS device, and Tooth Memo app on Android device were compared among 3 data recorders who entered patients’ information on decayed, missing, and filled permanent teeth (DMFT) index and community periodontal index (CPI), which were read aloud from the database of 103 patients by another dental personnel. The interobserver reliability of the 3 different data-entering procedures was evaluated by percent disagreement and kappa statistic values. Laypeople had access to the personal oral health record section of this app, and their satisfaction was evaluated through a Likert scale questionnaire. The satisfaction assessments for both sections of the Tooth Memo app involved the same set of questions on the app design, usage, and overall satisfaction.

**Results:**

Of the 103 dental records on DMFT and CPI, 5.2% (177/3399) data points were missing in the manual data entries, but no data on tooth status were missing in the Android and iOS methods. Complete CPI information was provided by all 3 methods. Transferring data from paper to computer took an average of 55 seconds per case. The manual method required 182 minutes more than the iOS or Android methods to clean the missing data and transfer and analyze the tooth status data of 103 patients. The users, that is, 109 fifth-year dental students and 134 laypeople, expressed high satisfaction with using the Tooth Memo app. The overall satisfaction with the oral health survey ranged between 3 and 10, with an average (SD) of 7.86 (1.46). The overall satisfaction with the personal oral health record ranged between 4 and 10, with an average (SD) of 8.09 (1.28).

**Conclusions:**

The Tooth Memo app was more efficacious than manual data entry for collecting data of oral health surveys. Dental personnel as well as general users reported high satisfaction when using this app.

## Introduction

Oral diseases such as tooth decay and gum disease remain prevalent in Thailand. According to the eighth National Oral Health Survey conducted in 2017 in Thailand [1], a significant percentage of the Thai population (31%-73.8%) had untreated caries, and less than 20% of the population was free of gum disease. The oral health survey is a crucial epidemiological method in dental health care that helps to understand the extent and prevalence of oral health problems [2]. The survey also provides preliminary data for planning projects to promote oral health locally and nationally [3]. Thailand conducts its national oral health survey every 5 years, recording several complex oral health measures according to the World Health Organization guidelines [4]. However, collecting data on oral health can be a time-consuming and error-prone process, leading to potential inaccuracies in diagnoses and treatment plans. Fortunately, advancements in mobile technology have made it possible to streamline data collection and analysis, providing a more efficient and reliable approach to oral health management.

Collecting data using pen-and-paper can lead to errors when transferring data to an electronic database due to poor legibility, unclear handwriting, smudged or fading ink, etc. Furthermore, manually entering data from a large number of participants into a database can be time-consuming. In today’s modern era, using technological devices in health care is becoming increasingly common [5-7]. Therefore, using an app on smartphones or tablets to input data may be more convenient than using computers and can increase the speed of analyzing and summarizing data [8,9], save time in transferring data from physical documents to electronic forms [10], and minimize paper expenses [10]. In the long term, data collected in mobile apps may help in the development of a database for research and advance the understanding of the state of oral health nationwide. Mobile apps for dental health care can be useful for improving access to oral care information, promoting preventive measures, simplifying appointment scheduling, monitoring the health of children and young people, and potentially offering features for virtual consultations or teledentistry [11,12]. The implementation of mobile health has the potential to enhance the delivery of health services [12]. Unfortunately, no mobile app is currently available for collecting oral health survey data. However, many mobile apps for oral health promotion aim to increase knowledge and promote healthy oral health behaviors [13].

The Oral Health Survey Mobile Application (OHSMA) [14] was created to collect data of oral health surveys. Unfortunately, OHSMA was only available on Android devices. iOS users could only access OHSMA through a web-based platform that required internet connectivity. Dental health professionals found the app inconvenient to use because of its limited availability. Due to these issues, the use of OHSMA was discontinued. A new offline-capable app would be more beneficial for digitizing oral health survey data. It is important to note that dental history plays a significant role in forensic identification. However, obtaining patient records can be challenging because these may be spread across different hospitals and clinics [15]. A dental history record of the general population could be a potential solution to this challenge. Additionally, an individual’s oral health record could provide better insight into their past treatments, which could help dental professionals plan future dental services. A possible solution to address the challenge of oral health care in the general population is to create an individual dental history record. This record could provide valuable information to dental professionals about an individual’s past treatments, enabling them to plan better dental services for the future. Furthermore, people can maintain a personal oral health record to remember past oral health events and share it with their dentists. It is also important to note that the general Thai population does not visit dentists regularly [16], and raising awareness of oral health concerns could help encourage more regular dental checkups.

Introducing Tooth Memo—the revolutionary mobile app designed to digitize oral health survey data and personal oral health information. Unlike OHSMA [14], its predecessor, Tooth Memo is an improved version and is compatible with both Android and iOS devices. Tooth Memo can be used offline, thereby making it very useful for conducting oral health surveys in areas with limited internet connectivity, especially in rural areas. This app offers several upgrades that can help health care workers to interpret the data more efficiently. For example, this app can calculate the mean decayed, missing, and filled permanent teeth (DMFT) index for all participants in each survey instantly and notify the health care worker if the examination is incomplete or complete. Tooth Memo has not only dental health surveys but also other survey forms for dental fluorosis and prosthesis status. The current version of the Tooth Memo app is designed to provide a more constructive experience for health care workers, making it easier for them to conduct oral health surveys and interpret the data more efficiently. This innovative app offers dental health professionals a convenient and user-friendly way to input data, thereby saving time and cutting down on paper expenses. Due to the addition of new design features, enhanced appearance, and increased functions in the Tooth Memo app compared to those in OHSMA, user satisfaction with this app was re-evaluated.

By providing dental health professionals with a reliable and efficient tool for data collection, Tooth Memo has the potential to improve the quality of dental care and promote better oral health outcomes for all. This study was conducted to evaluate the time efficiency, reliability, and user satisfaction of the Tooth Memo app.

## Methods

### Study Population and Methodology

This cross-sectional study compares the efficiency of 3 data collection methods for oral health surveys and explores the user satisfaction with the Tooth Memo app. The 3 methods for oral health survey data collection are (1) pen-and-paper (manual), (2) Tooth Memo app in iOS (iOS), and (3) Tooth Memo app in Android (Android). The Tooth Memo app was designed for 2 types of users: dental personnel who record oral health survey data and laypeople who record their own oral health information.

### Tooth Memo App

**Figure 1:**
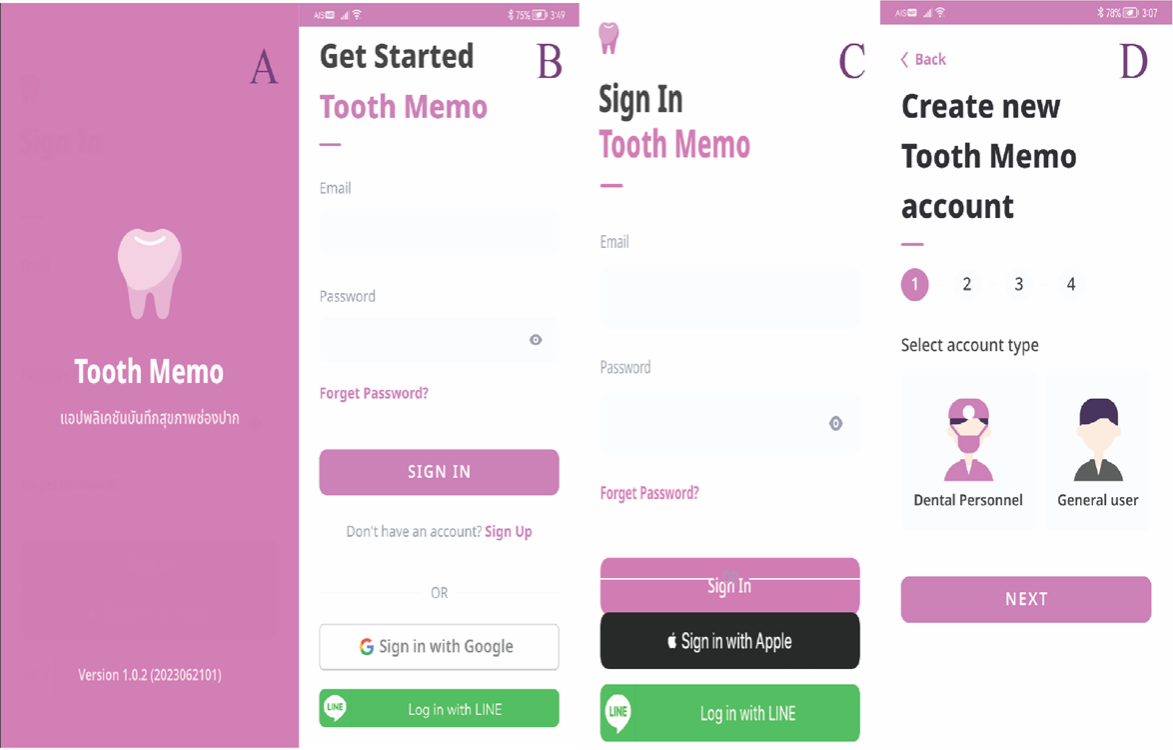
Screenshots of the (A) first page, (B) Android sign-in page, (C) iOS sign-in page, and (D) account selection in the Tooth Memo app.

The Tooth Memo app is designed for dental professionals to easily collect and analyze oral health survey data as well as manage personal oral health records. The Tooth Memo app is an improved version of OHSMA [14], in which the pitfalls or weaknesses of OHSMA have been addressed. This app is available for installation from the App Store or Play Store in both iOS and Android devices, respectively, and supports Thai or English language use based on the device setting. iPhone or iPod touch requires iOS 13.0 or later versions. Android phones require Android 6.0 and later versions. There are 2 account types in this app: (1) dental personnel and (2) general user (Figure 1).

**Figure 2:**
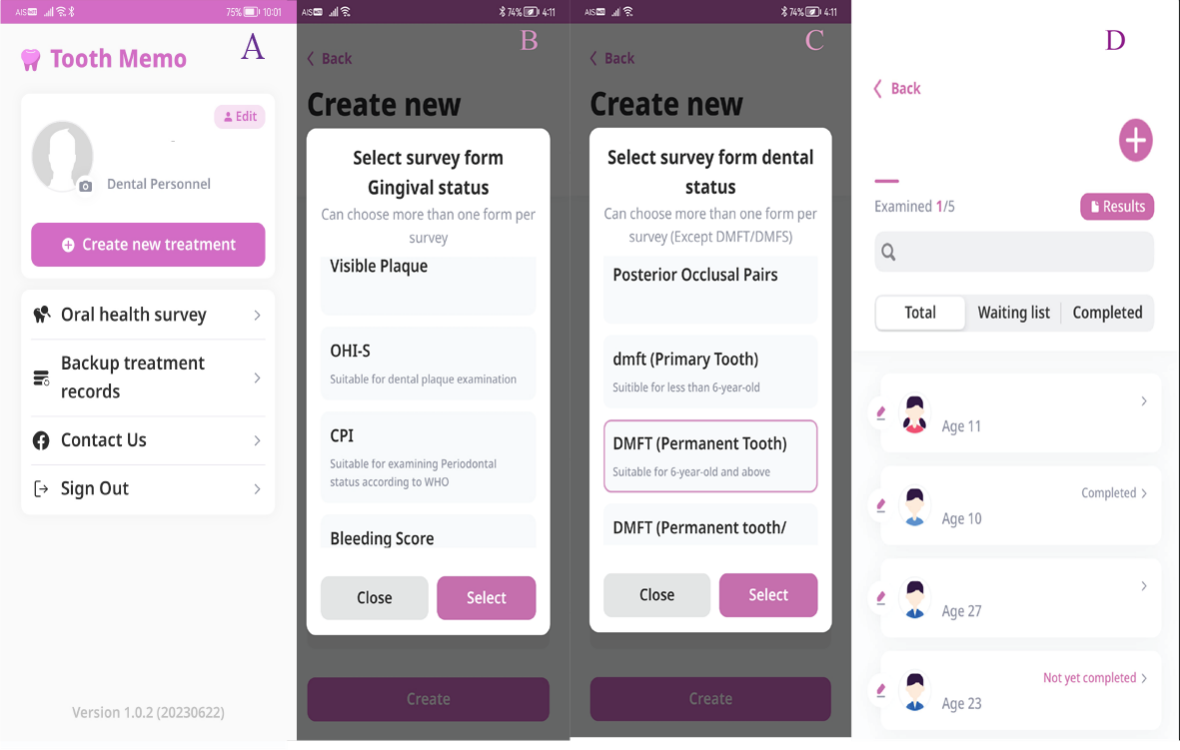
Screenshots of the oral health survey in the dental personnel feature: (A) first page, (B) gingival status forms, (C) dental status forms, and (D) list of participants.

The Tooth Memo app has 2 main sections. The first section, that is, oral health survey (Figure 2), allows dental professionals to record the oral health survey data according to the fourth and fifth editions of World Health Organization Oral Health Surveys-Basic Methods [4,17]. Dental personnel can record dentition status [17]; prosthetic needs; number of posterior occlusal pairs; DMFT index; and the decayed, missing, and filled permanent surfaces (DMFS) index. Gingival health can also be recorded using the community periodontal index (CPI) [4] and simplified oral hygiene index [18]. Tooth Memo provides a function for uploading individual characteristics, including name, gender, and age of each survey participant, for convenient usage. Dental personnel can collect the surveyed data on each device with or without internet connectivity. The recorded data will be retained in each data-entering device, and a summary report can be created and exported as an Excel spreadsheet. The user can also upload the name list of the sample before the survey. There are notices for incomplete data collection.

**Figure 3:**
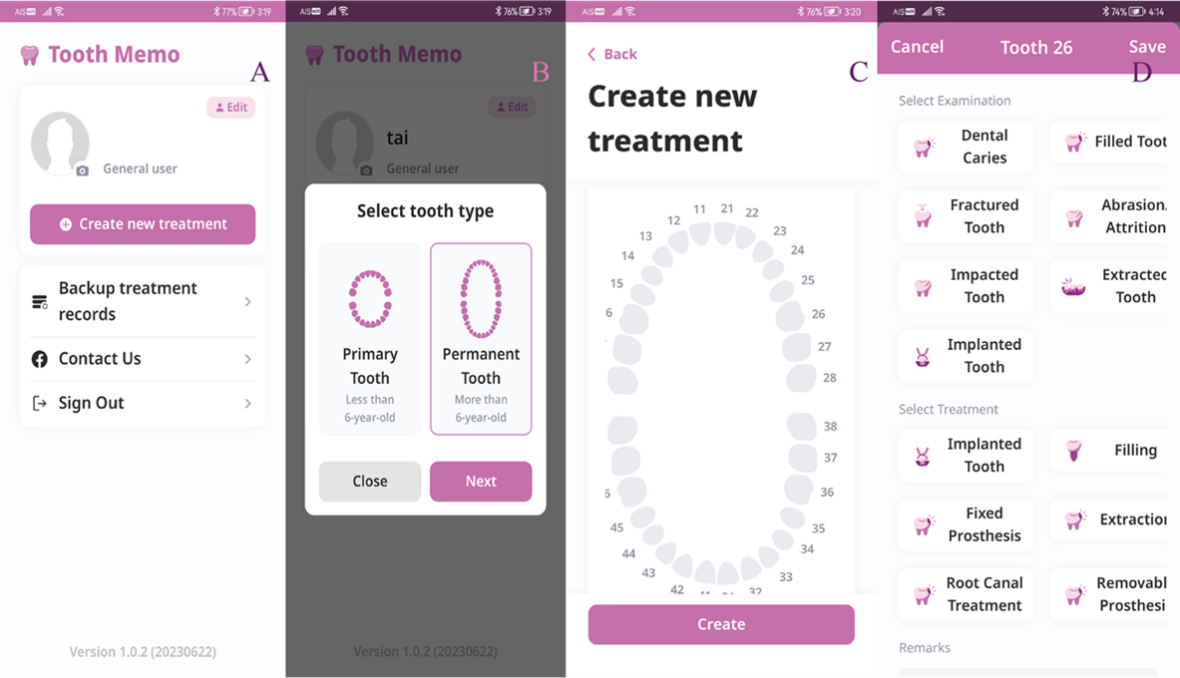
Screenshots of the personal oral health record in the general user feature: (A) first page, (B) tooth type selection, (C) dental chart for data entry, and (D) status and treatment for each tooth.

The second section, that is, personal oral health record (Figure 3), allows users to manage their oral health status and treatment for each tooth. Different charts are available for primary and permanent dentition, and Tooth Memo can record the treatment date and 1 image on each device. Dental professionals can access both sections, while laypeople can only access the personal oral health record section.

### Capability Assessment

The study aims to test the interobserver reliability of 3 different data-entering procedures, namely pen-and-paper (manual), iOS app (iOS), and Android app (Android), using the dentition status (DMFT) and gingival status (CPI) of 103 patients from the database of the Department of Community Dentistry, Chulalongkorn University. For each data-entering procedure, 3 data recorders entered each patient’s information on DMFT and CPI, while a dental personnel read out this information aloud simultaneously from the database. The data recording by pen-and-paper was transferred to the computer, and the timing for entering and summarizing the data in the manual method was recorded. The interobserver reliability was assessed using test-retest reliability (κ) and percent agreement among different methods.

### Satisfaction Assessment

#### Oral Health Survey

1. Dental students collected oral health survey data through the Tooth Memo app during their clinical work, and their satisfaction with the app was evaluated using a questionnaire. These students had no experience with other apps for oral health surveys, although they are familiar with mobile apps. Tooth Memo is their first app for oral health surveys. Each dental student examined 4-8 patients during their clinical work.
2. In this study, all fifth-year dental students of the 2022 academic year from the Department of Community Dentistry, Faculty of Dentistry, Chulalongkorn University, were recruited.

#### Personal Oral Health Record

1. Laypeople who can read Thai and voluntarily participated in this study recorded their dental status and treatments in Tooth Memo, and their satisfaction was evaluated after using the app.
2. The minimum sample size for this study was estimated using the GPower 3.1 Program [19] for a 1-sample case. The 2-sided *t* test for difference of means from constant (1-sample case) was used for calculating the required sample size by given α (.05), power (.95), and effect size (0.318) [14]. The suggested total sample size was 131.

#### Overall Satisfaction

User satisfaction was evaluated with a newly developed self-administered questionnaire in Thai via a Google form. The questionnaire had undergone a thorough revision process based on the findings of a previous study [14]. Modifications were made to improve the clarity and relevance of the questions. The revised questionnaire was then pilot-tested in a sample group to evaluate its effectiveness in capturing the intended information. The feedback from the pilot test was used to further refine the questionnaire and ensure that the questions were clear, concise, and easy to understand. The questionnaire had 2 parts, with identical questions for each section of the Tooth Memo app (personal oral health record and oral health survey). Satisfaction with the design and usage of Tooth Memo and the overall satisfaction were evaluated. The satisfaction questions on the design of Tooth Memo were related to the font style, size, and color, appropriate and sufficient content in each page, continuity in content across pages, and the channel for consultation if a problem occurs. The satisfaction questions on the usage of Tooth Memo included registration, recording the data, summary and report, searching the recorded data, and loading speed. Each part of the questionnaire had nine 5-point Likert scale questions concerning users’ satisfaction. The scores ranged from 1 to 5 (1=least appropriate, 2=less appropriate, 3=moderately appropriate, 4=highly appropriate, and 5=most appropriate). Additionally, there was an 11-point rating scale (0-10 points) concerning overall satisfaction.

### Data Analysis

#### Capability Assessment

We compared the errors incurred and time taken for data entry and data summarizing among the 3 methods. Interrater reliability was analyzed using Cohen kappa [20], Fleiss kappa, and percent disagreement [21]. This study follows the STROBE (Strengthening the Reporting of Observational Studies in Epidemiology) guidelines [22] for reporting.

#### Satisfaction Assessment

User satisfaction was analyzed using SPSS software (version 29.0; IBM Corp) through descriptive statistics, mean, standard deviation, frequency, and percentage. The proportion of each score among the satisfaction questions was analyzed using frequency and percentage.

### Ethics Approval

The study protocol was approved by the Human Research Ethics Committee of the Faculty of Dentistry at Chulalongkorn University (HREC-DCU 2023-001) before the study began. Consent was obtained from the Department of Community Dentistry, Chulalongkorn University, to access data for the research. The participants were provided with a clear information sheet outlining the project’s aims, and they were free to choose whether they wanted to participate in this study. The questionnaires were designed to be anonymous. The study participants were informed that they could withdraw from the research at any time and were not obligated to complete the questionnaire. Completing and submitting the questionnaire were considered as participants’ consent to participate in this study. As compensation for their time, a toothbrush was given to the participants.

## Results

In 103 dental records, 5.2% (177/3399) data points were missing in the manual entries. However, the Android and iOS methods showed no missing data on tooth status. It is worth noting that complete CPI information was provided by all 3 methods.

### Capability Assessment

Our findings showed that analyzing the dmft/DMFT (decayed, missing, and filled primary teeth/decayed, missing, and filled permanent teeth) data on iOS and Android platforms takes less than a minute. The app performed the dmft/DMFT calculations; so, the time required for data analysis is negligible. In the manual method, transferring data from paper records to the computer took 95 minutes, averaging approximately 55 seconds for each case. Additionally, cleaning up missing data consumed 63 minutes, while the analysis of DMFT required an additional 25 minutes. As a result, the manual method took 182 more minutes than the iOS or Android method to transfer data to the computer and analyze the data of 103 patients. The summary of the time taken for each step in the data entry methods can be found in Multimedia Appendix 1. Of the 103 patients, 42.7% (44/103) were females and 7-10 years of age, with an average age of 7.40 (SD 0.61) years. The dmft and DMFT of 103 patients were 5.32 and 0.29, respectively. dt/DT (decayed primary teeth/decayed permanent teeth), mt/MT (missing primary teeth/missing permanent teeth), and ft/FT (filled primary teeth/filled permanent teeth) were 4.83/0.23, 0.18/0, and 0.31/0.06, respectively. Moreover, 5.8% (6/103) of the patients had healthy gingival status (CPI score = 0) and 11.6% (12/103) needed scaling (CPI score = 2). The overall Fleiss kappa was 0.93 among the 3 methods, and Table 1 shows the Cohen kappa and percent disagreement among the 3 methods. The Tooth Memo app in both iOS and Android platforms showed superior results compared to those obtained using pen and paper. The iOS and Android versions of the app recorded complete data with no missing information. Additionally, there was a lower percentage of disagreement between the data collected using the app on the iOS and Android platforms.

**Table 1:** Cohen kappa values and percent disagreement between different methods of oral health survey data recording (n=3399).

Methods	Manual versus iOS	Manual versus Android	iOS versus Android
Disagreement, n (%)	269 (7.9)	243 (7.2)	59 (1.7)
Cohen kappa	0.90	0.91	0.98

### Satisfaction Assessment

**Figure 4:**
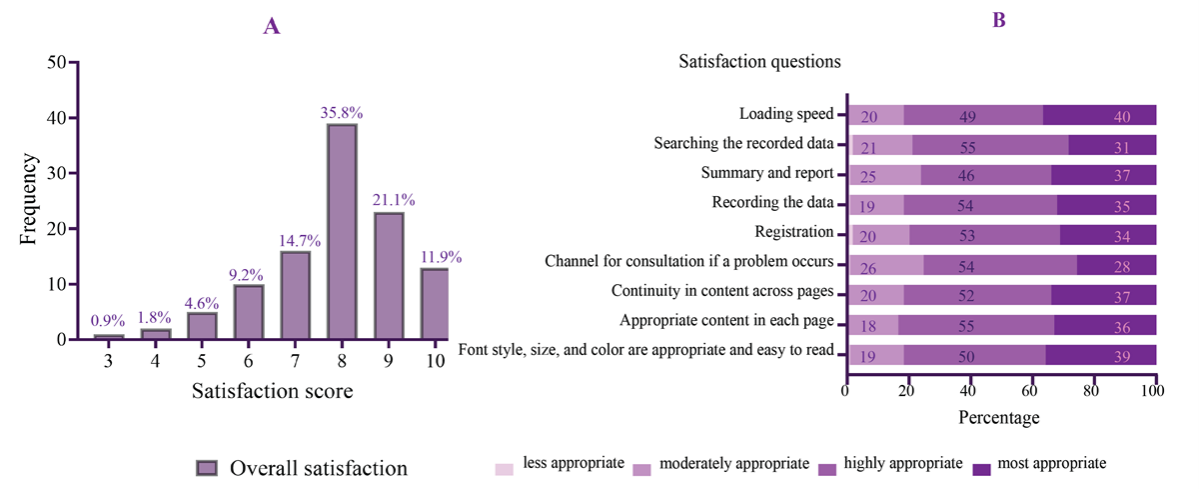
(A) Overall satisfaction and (B) percentage of dental personnel who indicated their satisfaction level (less appropriate, moderately appropriate, highly appropriate, and most appropriate) for each question in the oral health survey section.

In this study, 109 fifth-year dental students aged 21-23 years collected oral health survey data in Tooth Memo during their clinical work. Of these students, 62.4% (68/109) were males. The overall satisfaction of the users ranged between 3 and 10, with an average (SD) of 7.86 (1.46) (Figure 4A). The users were generally satisfied with the app’s design and usage, with average scores ranging from 4.0 to 4.18. The average design satisfaction scores ranged from 4.0 to 4.17, while the average usage satisfaction scores ranged from 4.06 to 4.18. The medians of the design and usage satisfaction scores were both 4. The design satisfaction scores of each question ranged from 2 to 5 (Table 2). Most fifth-year dental students used tablets rather than mobile phones, and most of them used the iOS platform. The proportions of satisfaction levels for each question are presented in Figure 4B.

**Table 2:** Descriptive statistics of the satisfaction levels of the users who used the Tooth Memo app.

		Oral health survey (n=109)		Personal oral health record (n=134)	
		Mean (SD)	Range	Mean (SD)	Range
**Design**					
	Font style, size, and color are appropriate and easy to read	4.17 (0.74)	2-5	4.16 (0.69)	2-5
	Appropriate content in each page	4.17 (0.69)	3-5	4.15 (0.68)	2-5
	Continuity in content across pages	4.16 (0.71)	3-5	4.12 (0.66)	3-5
	Channel for consultation if a problem occurs	4.00 (0.73)	2-5	4.02 (0.71)	2-5
**Usage**					
	Registration	4.09 (0.75)	2-5	4.15 (0.68)	2-5
	Recording the data	4.13 (0.72)	2-5	4.07 (0.69)	2-5
	Summary and report	4.09 (0.78)	2-5	4.06 (0.63)	3-5
	Searching the recorded data	4.06 (0.74)	2-5	4.14 (0.67)	3-5
	Loading speed	4.18 (0.72)	3-5	4.12 (0.72)	2-5
Overall satisfaction		7.86 (1.46)	3-10	8.09 (1.28)	4-10

**Figure 5:**
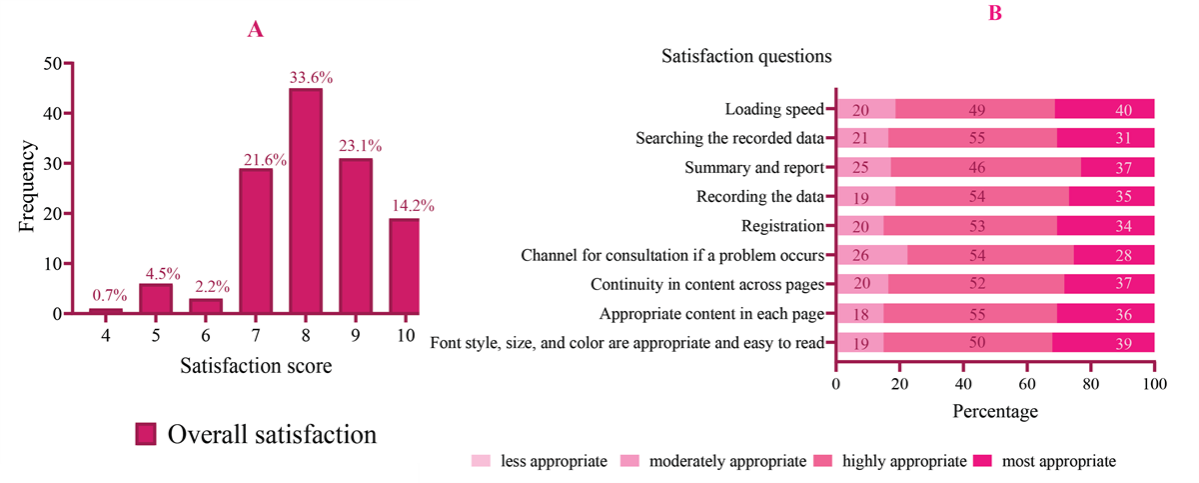
(A) Overall satisfaction score and (B) percentage of laypeople who indicated their satisfaction level (less appropriate, moderately appropriate, highly appropriate, and most appropriate) for each question in the personal oral health record section.

In our study, 134 laypeople reported their satisfaction after using the personal oral health record section of Tooth Memo. The users were 15-80 years old, and 26.9% (36/134) were males. Equal numbers of laypeople used the iOS and Android platforms to access the personal oral health record section of the Tooth Memo app. The overall satisfaction scores ranged between 4 and 10, with an average (SD) of 8.09 (1.28) (Figure 5A). The laypeople were generally satisfied with the app’s design and usage, with average scores ranging from 4.02 to 4.15. The average design satisfaction scores ranged from 4.02 to 4.16, while the average usage satisfaction scores ranged from 4.06 to 4.15. The medians of the design and usage satisfaction scores were both 4. The design satisfaction scores of each question ranged from 2 to 5, and the proportions of different satisfaction levels for each question are shown in Figure 5B.

## Discussion

The Tooth Memo app demonstrated good reliability, high time efficiency, and high user satisfaction. Tooth Memo was developed based on the experience of OHSMA [14] and interviews with stakeholders. The Tooth Memo app has various forms for different indexes in the oral health survey. User-friendly functions were developed, such as operating without internet connectivity, a button for patients with no tooth decay, notification of completeness of data collection, and provision of only the necessary active buttons. The recorded data will be stored in each device. All recorded data and the analysis results can be exported into an Excel form.

Tooth Memo provides only the necessary active buttons at each data entry point to minimize data entry mistakes. Screenshots of the active buttons for each data entry point, that is, tooth status, treatment need of each tooth, and gingival status are shown in Multimedia Appendix 2. During the design phase, there was a concern regarding the size of the button. Eventually, it was decided that the button should be large enough to accommodate data collection. In mobile devices, the button size is 8×8 millimeters, while in tablets, it is 12×12 millimeters, which makes data entry easier. For process simplicity, registration is only required once for each device during the first use. These points were not included in the satisfaction assessment but were open for comments at the end of the survey. However, no comments were received regarding these points.

This app can benefit researchers, as it has various forms for recording the gingival and dental status. Screenshots of the DMFS, active buttons, and code explanations are given in Multimedia Appendix 3. The dmfs/DMFS (decayed, missing, and filled primary surfaces/DMFS) with the explanation for each code is given in Multimedia Appendix 3. There are functions for reducing the time for data collection, such as the “no tooth decay” button for patients without tooth decay (Multimedia Appendix 3) and the active button’s automatic move after each data entry. The participant name list can be uploaded in the app before going to the site for data collection so that the time spent in filling those data at the site can be saved. Moreover, Tooth Memo can display the data collection status of each participant (Figure 2D). The users who participated in the oral health survey were fifth-year dental students. Although their familiarity with using mobile apps may have influenced their satisfaction results compared to other age groups, they were able to provide valuable insights about the user interface and suggest areas for improvement.

Additionally, Tooth Memo can be an individual’s personal dental history recorder. The tooth status and dental treatment of each tooth can be recorded along with the date of examination and treatment, especially endodontic treatment, which needs further dental procedures. The scatter data of each person can be gathered in their device along with the date and place. The recorded data will benefit future treatment plans or even forensic purposes. The accuracy of recording can be enhanced if each individual can ask the dental personnel about their tooth status and treatment after their dental visit.

This study examines the capability of the “Tooth Memo” for collecting oral health survey data. The time spent on data entering was controlled by simultaneous data entry using the 3 methods. The difference between the time taken in the manual method and the time taken in the iOS or Android methods was attributed to the time spent for transferring data into a computer, cleaning the missing data, and analyzing the data. The manual method takes 182 more minutes than the iOS or Android methods to transfer data to the computer and analyze the data of 103 patients. It is interesting to note that the iOS and Android platforms are much faster when analyzing dmft/DMFT data, taking less than a minute to perform the calculations. However, the manual method takes significantly longer, with data transfer from paper records to the computer, cleaning up missing data, and DMFT analysis.

Our results indicated more missing data in the manual data-entering method than in the iOS and Android methods. The manual method required time for entering data in an average of 1 minute per case and more time for cleaning and analyzing the data, indicating that digitalized data in Tooth Memo could increase efficiency by decreasing the time taken and the errors that might occur while transferring data into the computer. The manual data-entering method required 2 steps for transferring data, thereby increasing the chance of error. This finding was supported by the high disagreement and low kappa statistic values between the manual method and the iOS/Android methods, while the iOS and Android methods showed less disagreement and high kappa statistic values. Less disagreement could result from the active buttons provided for each data entry point being the only possible codes. The impossible codes for each data entering point will be inactive. The manual method required 182 more minutes to achieve the result of the data analysis in this small survey, while Tooth Memo could save time and budget in transferring data in around 1 minute per case. These findings show that using the Tooth Memo app can be efficient and save time and budget for a more extensive survey.

All users indicated high satisfaction with the design and usage of Tooth Memo. Responses to all satisfaction questions indicated high user satisfaction for both sections of Tooth Memo. We did not compare the satisfaction rates between the manual method and the iOS/Android method because a previous study [14] had already shown higher satisfaction with the mobile app than the pen-and-paper method.

As Tooth Memo is a newly developed mobile app, users may not be familiar with the app. However, app unfamiliarity might be present only in the learning period. Nevertheless, some users did indicate low satisfaction with both sections of Tooth Memo. Some comments for improvement were related to enhancing the design by adding new interactive and attractive user-friendly features and increasing the stability of the app.

We do not have any other app to compare with our app. Our app is the first of its kind to collect oral health survey data and provide personalized individual oral health records. We are aware of other data collection apps for health [5,6,12,23-28] and non–health data [29-31], but there is no specific app like ours. There was only 1 app [14] for oral health survey data collection, but it was discontinued due to its inconvenience. Therefore, we were unable to make any comparisons. Interestingly, there are many apps [12,13,32-45] for oral health promotion that aim to promote knowledge and behaviors related to oral health; there is also an artificial intelligence app that can detect dental caries [43]. Our app follows the standard format used by most apps such as banking or other utility apps.

As per the feedback provided by some users, there are some areas for improvement in Tooth Memo’s oral health survey section. Although this app has a user-friendly interface and can record the data of a large number of participants in each survey and can record many surveys, there are some disadvantages that need to be considered. The log-in system is unstable on the new version of iOS, data files cannot be exported on certain devices, and there is no interface for iPad users. Additionally, data cannot be shared among users with the same account, and there were some miscalculations in the summary data. To improve the system, these issues need to be addressed. Despite these drawbacks, the survey provides explanations for each code, collects both dental and gingival status, and provides a summary of each participant’s data, along with the data collection status for each participant. Similar to other mobile apps such as the electronic medical records app [46] and Ru Tan Ya app [47], Tooth Memo enhances the effectiveness of self-care, improves continuity of care, simplifies data collection, decreases overhead costs, reduces mortality in various kinds of patients, saves time for professionals, and helps to avoid transcription errors.

The Tooth Memo mobile app is an innovative tool that empowers individuals to take control of their oral health. By providing accurate data on previous treatments, this app helps users make informed decisions about their dental care. It also serves as a helpful reminder for any untreated teeth that require attention, ensuring that users stay on top of their oral hygiene. Additionally, this app offers a convenient mobile record of an individual’s dental health history, which can be a valuable resource for those who do not regularly visit the dentist. Overall, this app is an excellent resource for anyone looking to improve their oral health and increase their oral health literacy. But Tooth Memo is not just a game changer for data collection. This app also has the potential to significantly aid in forensic identification, as a record of an individual’s oral health history can provide valuable insight into their past treatments and dental services. Furthermore, having a record of the dental history of the general population can make it easier for the health care system to obtain patient records, which are often scattered across different hospitals and clinics.

There are several mobile apps for data collection [29-31,48] for various types of research. These apps incur a lower cost and are more effective for data collection than the pen-and-paper method similar to the findings reported in our study. Digital data collection can provide more data security, accountability, and accuracy, save time, and even reduce costs.

One limitation of this study is that the Tooth Memo app is currently in the development stage and is not widely used. Therefore, we cannot conduct a long-term study yet to explore and analyze the results from different groups of participants. However, once this app is launched to the public, we will be able to gather more data and make necessary improvements to provide an effective and user-friendly app that meets the needs of the users. Since this is the first app for oral health survey data collection and personalizing individual dental history, there is no comparable information to compare the satisfaction levels of users. Nonetheless, the feedback provided by the users was useful for further improvement.

The Tooth Memo app significantly reduces the time and effort required for data entry and analysis. This app also had fewer missing data points and lesser disagreement between data entry platforms. Users expressed high levels of satisfaction with the app’s design and functionality.

## Appendix

Multimedia Appendix 1 Summary of the time taken for each step in the data recording.

Multimedia Appendix 2 Screenshots of the active buttons for each data entry point: (A) tooth status, (B) treatment need of each tooth, and (C) gingival status.

Multimedia Appendix 3 Screenshots of (A) decayed, missing, and filled surfaces; (B) active buttons; and (C) code explanation.

## Abbreviations

CPI: community periodontal index
DMFS: decayed, missing, and filled permanent surfaces
dmfs: decayed, missing, and filled primary surfaces
DMFT: decayed, missing, and filled permanent teeth
dmft: decayed, missing, and filled primary teeth
dt/DT: decayed primary teeth/decayed permanent teeth
ft/FT: filled primary teeth/filled permanent teeth
mt/MT: missing primary teeth/missing permanent teeth
OHSMA: Oral Health Survey Mobile Application
STROBE: Strengthening the Reporting of Observational Studies in Epidemiology

## Data Availability

The data sets generated during and analyzed during this study are available from the corresponding author on reasonable request.
