# Supplementary material for: Time Efficiency, Reliability, and User Satisfaction of the Tooth Memo App for Recording Oral Health Information: Cross-Sectional Questionnaire Study
Source: JMIR Form Res. 2024 Apr 10;8:e56143. doi: 10.2196/56143 (PMC11043928; doi:10.2196/56143)
Supplement: Multimedia Appendix 1 [file formative_v8i1e56143_app1.pptx]

## Slide 1
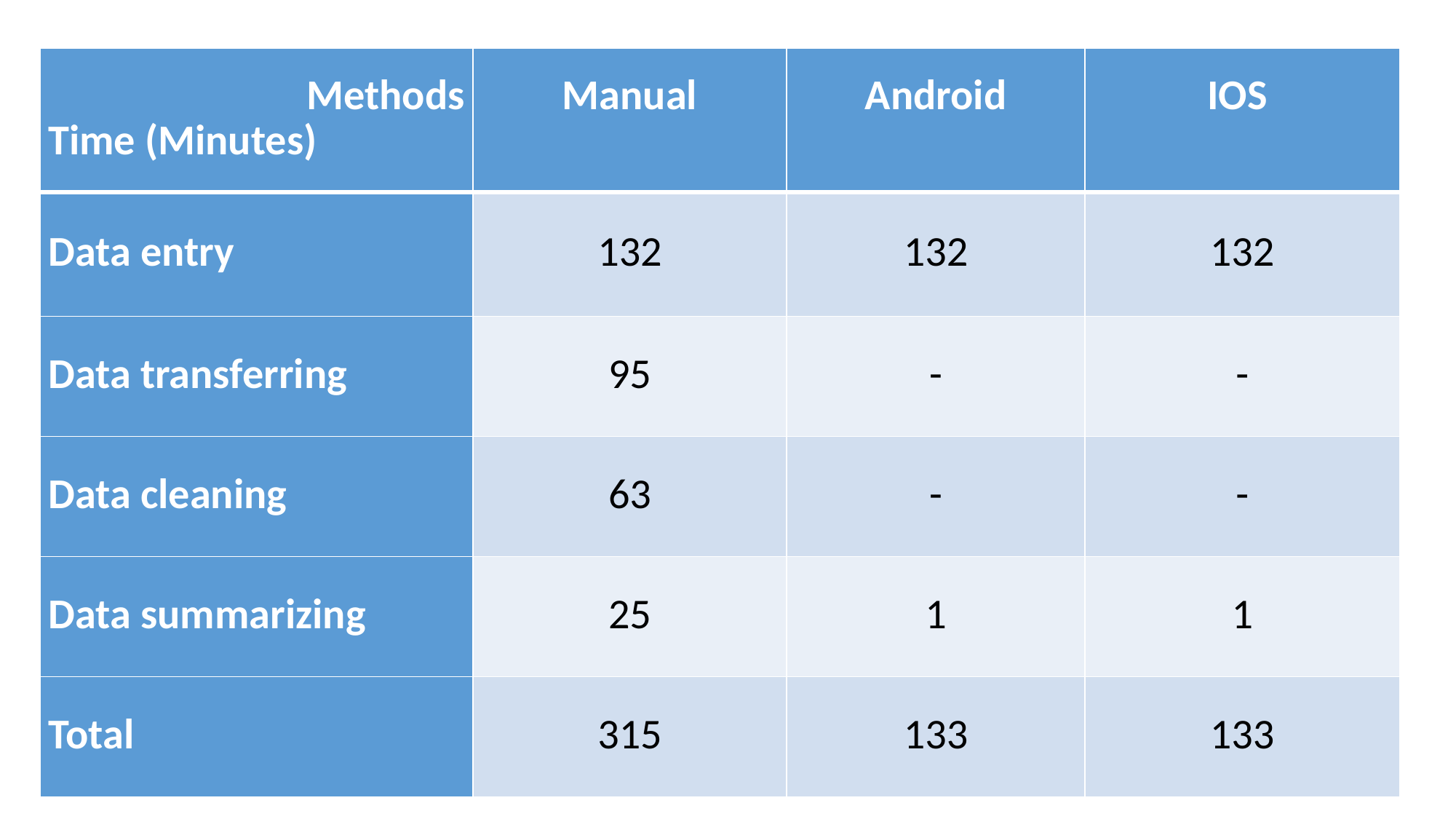

| Methods Time (Minutes) | Manual | Android | IOS |
| --- | --- | --- | --- |
| Data entry | 132 | 132 | 132 |
| Data transferring | 95 | - | - |
| Data cleaning | 63 | - | - |
| Data summarizing | 25 | 1 | 1 |
| Total | 315 | 133 | 133 |
