# Supplementary figures and images for: Time Efficiency, Reliability, and User Satisfaction of the Tooth Memo App for Recording Oral Health Information: Cross-Sectional Questionnaire Study
Source: JMIR Form Res. 2024 Apr 10;8:e56143. doi: 10.2196/56143 (PMC11043928; doi:10.2196/56143)

## Slide 1
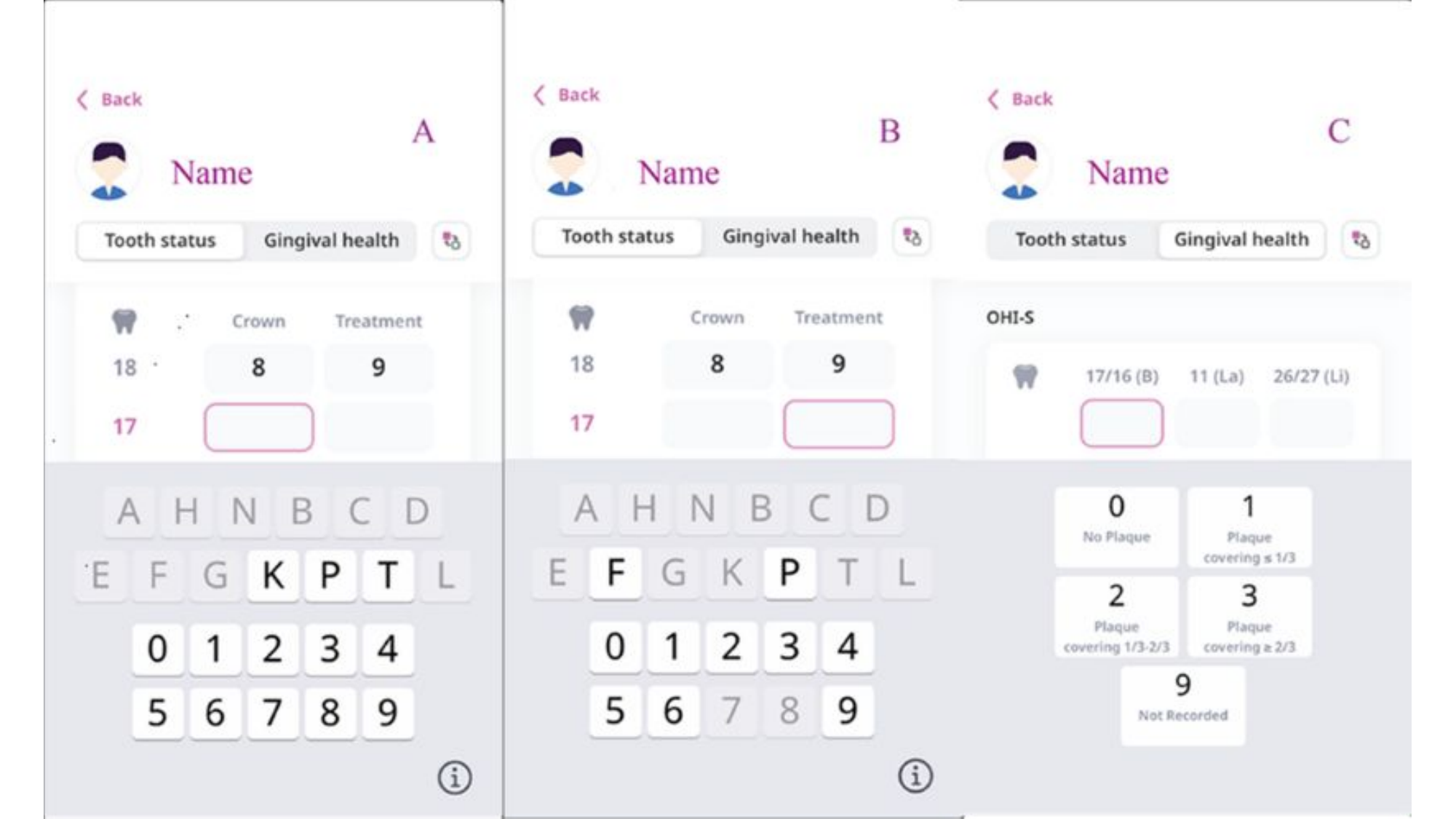

#

Supplement: Multimedia Appendix 2 [file formative_v8i1e56143_app2.pptx]

## Slide 1
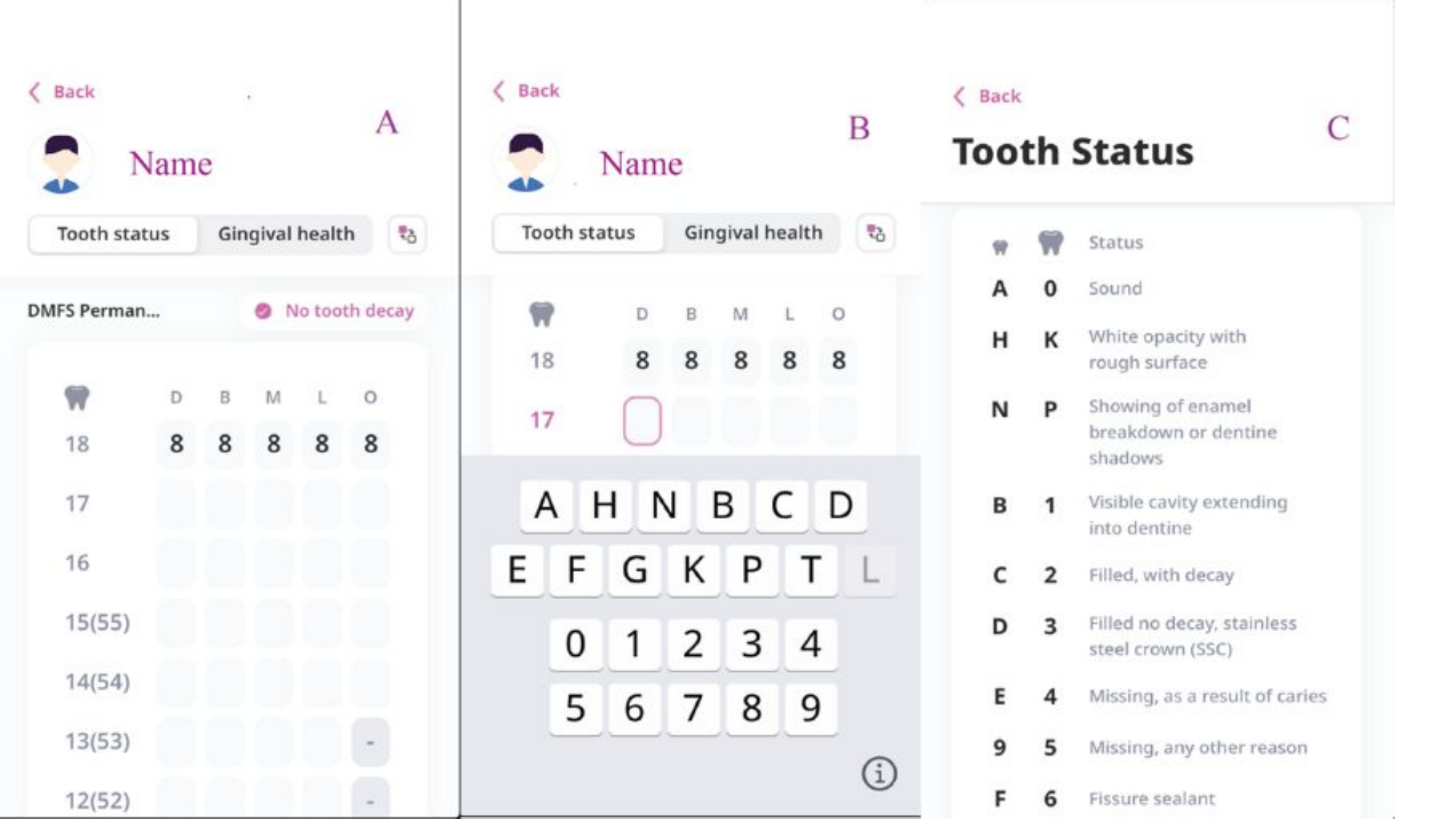

#

Supplement: Multimedia Appendix 3 [file formative_v8i1e56143_app3.pptx]
